# From Structure to Aesthetics: The Importance of Nasal Bone Thickness in Rhinoplasty Planning

**DOI:** 10.3390/jcm15124801

**Published:** 2026-06-20

**Authors:** Marcin Jadczak, Michał Kaczmarczyk, Paweł Rozbicki, Dariusz Jurkiewicz, Sandra Krzywdzińska

**Affiliations:** Department of Otolaryngology with Division of Cranio-Maxillo-Facial Surgery, Military Institute of Medicine-National Research Institute, 04-141 Warsaw, Poland; mkaczmarczyk@wim.mil.pl (M.K.); djurkiewicz@wim.mil.pl (D.J.)

**Keywords:** rhinoseptoplasty, osteotomies, nasal bones

## Abstract

**Background**: Correct performance of rhinoseptoplasty often requires osteotomy, the effectiveness and safety of which depend on precise knowledge of nasal anatomy, particularly the shape, length, and thickness of the nasal bones. **Aim**: The aim of this study was to assess the morphometry of the nasal bone and nasal pyramid in adult patients based on three-dimensional computed tomography (CT) using Slicer 3D software. **Materials and Methods**: A retrospective analysis was performed on data from 87 patients (44 women and 43 men; mean age 50.64 SD ±16.7 years) who underwent head CT between 1 January 2024 and 31 December 2025 because of trauma (to exclude intracranial hemorrhage) or dizziness (to diagnose central causes). **Results**: The comparative analysis demonstrated statistically significant differences in the dimensions of the nasal bony structures between women and men, with women showing lower values for all assessed parameters. For point 1L, the mean value was 6.29 in women compared with 8.07 in men (*p* = 0.0056). For point 4L, the values were 2.07 vs. 2.55, respectively (*p* = 0.009), whereas for point 5L they were 1.52 vs. 1.66 (*p* = 0.01). A similar relationship was also noted on the right side: for point 1R, the values were 5.95 vs. 8.02 (*p* = 0.03), and for point 5R they were 1.54 vs. 1.89 (*p* = 0.03), confirming consistently smaller dimensions of the bony structures in women in the analyzed study group. **Conclusions**: The obtained results may have important practical significance in planning osteotomy during rhinoseptoplasty procedures, enabling a more precise and safer surgical procedure.

## 1. Introduction

Rhinoseptoplasty is one of the most commonly performed procedures in both plastic surgery and otolaryngology, combining aesthetic refinement with functional correction of the nose [1]. A critical stage of this operation is osteotomy, defined as the controlled transection of the nasal bones to enable their repositioning and achieve the desired contour of the nasal pyramid. This step is indicated, among others, for narrowing a wide nasal dorsum, closing open roof deformities following hump removal and correcting nasal deviation. Despite its importance, osteotomy remains the most traumatic and least predictable component of rhinoplasty [2]. The precision with which it is performed has a direct impact on the final surgical outcome, influencing both aesthetic results and nasal airway patency.

The choice of the incision site, surgical technique, instruments and force required to perform osteotomy depends largely on the individual anatomical characteristics of the patient and especially on the thickness and density of the nasal bones. Thicker and more compact bones may require greater force when using surgical instruments such as osteotomes or the use of more precise instruments (for example, piezoelectric instruments). Conversely, thinner bones are more susceptible to uncontrolled fractures, which is associated with a higher risk of complications, asymmetry or ultimately an unsatisfactory final result of the performed operation. For this reason, knowledge of the morphometric parameters of the nasal bones (their thickness, length and width) may significantly support surgical planning and improve the safety and effectiveness of the procedure [3].

Despite the widespread performance of rhinoseptoplasty, there remains a relative paucity of quantitative data in the literature regarding the dimensions and density of nasal bones in the adult general population, particularly those derived from three-dimensional reconstructions of computed tomography (CT) imaging.

The aim of the present study is to enhance the understanding of nasal osteotomy, specifically bone transection during rhinoplasty through a comprehensive analysis of nasal surface aesthetics, anatomical considerations and surgical techniques. Furthermore, this study seeks to evaluate the morphology of the nasal bones and the nasal pyramid in adult patients using CT-based three-dimensional reconstructions with particular emphasis on parameters relevant to osteotomy in rhinoseptoplasty.

## 2. Materials and Methods

**Figure 1:**
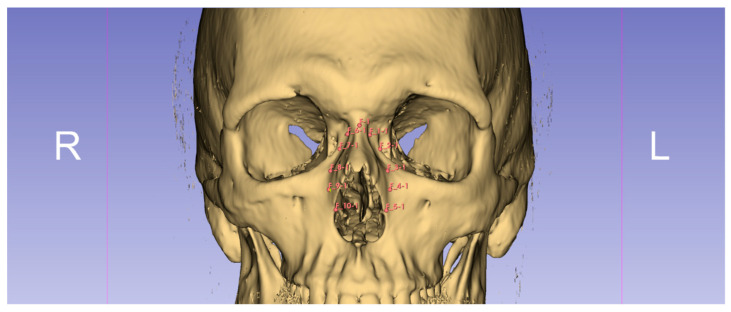
The figure shows an orthographic frontal view with marked osteotomy points.

**Figure 2:**
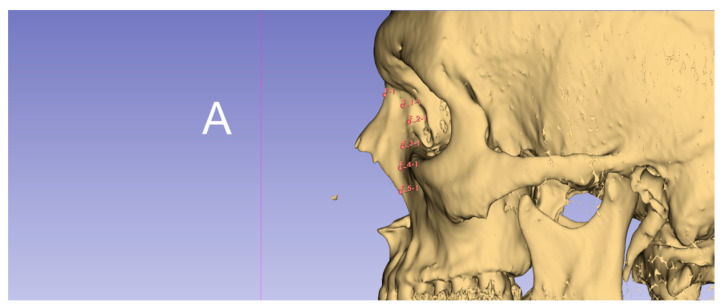
The figure shows an orthographic lateral view with marked osteotomy points. Point F-1 was defined as the Radix. Points F-1-1, F2-1, F3-1, F4-1, F5-1, for clearer presentation of the results, were presented as 1-5L (corresponding to the left side), whereas points F6-1, F7-1, F8-1, F9-1, F10-1 were presented as 1-5R (corresponding to the right side).

In this retrospective study, data from 87 patients (44 women and 43 men; mean age 50.64 SD ± 16.7 years) who underwent head computed tomography (CT) at the Military Institute Of Medicine National-Research Institute between 1 January 2024 and 31 December 2025 were analyzed. Imaging studies were performed for two main clinical indications, to exclude intracranial hemorrhage after head trauma and to diagnose dizziness, with the aim of excluding central causes. Approval was obtained from the Bioethics Committee of the Military Institute of Medicine-National Research Institute, No. 38/25 dated 25 June 2025. Head CT examinations were analyzed in which three-dimensional reconstruction of the bony structures of the facial skeleton was performed using Slicer 3D software(version 5.6.1), with particular attention to the dimensions and thickness of the nasal bones (Figure 1 and Figure 2).

3D Slicer software version 5.6.1 was used for image segmentation, three-dimensional reconstruction, and all measurements. CT datasets were acquired using a standard paranasal sinus CT protocol with a slice thickness of 0.625 mm and an in-plane resolution of 0.398 × 0.398 mm.

Prior to segmentation, all CT scans were aligned in the sagittal, coronal and axial planes to standardize image orientation and reduce measurement errors. Osseous structures were segmented using the automated thresholding method available in the Segment Editor module. A threshold range of 100–3000 HU was applied to isolate bone tissue. Following segmentation, a three dimensional model of the nasal bones was generated.

Osteotomy landmarks corresponding to the planned osteotomy sites were placed on the 3D bone model by one of two experienced rhinologists and confirmed by the rest of the team according to a predefined measurement protocol. The landmark coordinates were subsequently converted into corresponding segments and volumetric and radiodensity data were extracted automatically using the Segment Statistics module.

For each osteotomy point, the following parameters were obtained: segmented volume (mm^3^), mean Hounsfield Unit (HU), and median Hounsfield Unit (HU). Bone thickness measurements were performed at all predefined osteotomy points. Thickness was defined as the shortest distance between the external and internal cortical surfaces of the nasal bone measured along a line perpendicular to the local bone surface.

For points 1L and 1R located in close proximity to the nasion, measurements were obtained in the sagittal plane, corresponding to the anticipated direction of osteotomy forces. The starting point was placed on the external cortical surface of the nasal bone, whereas the endpoint was positioned on the internal cortical surface, extending to the most external aspect of the agger nasi cell when present.

For the remaining osteotomy points, measurements were obtained in the coronal plane using the same methodology. This orientation was selected to correspond with the local curvature of the nasal bones and the expected direction of force transmission during osteotomy.

Selected fragments of the manuscript were translated from Polish into English using a generative AI tool. All translated content was subsequently reviewed and edited by the authors.

Adult patients (≥18 years), irrespective of sex, were eligible for inclusion provided they had no prior history of rhinoseptoplasty or orthognathic surgery, no documented nasal bone fractures and no congenital or genetic craniofacial anomalies (e.g., cleft lip and palate) that could influence craniofacial morphology.

Exclusion criteria comprised age < 18 years, presence of malignancy, tumors or soft-tissue masses within the craniofacial region, prior radiotherapy to this area, poor-quality CT scans, imaging artifacts or incomplete radiological data preventing accurate morphometric assessment, previous nasal or sinonasal surgical procedures, a history of facial trauma or nasal bone fractures and any genetic or developmental conditions affecting the structure of the nasal or craniofacial skeleton.

### Statistical Analysis

After tabulating the data obtained from computed tomography images, the study population was evaluated according to sex, age and mean bone thickness at the points of interest (POIs). Next, the presence of a normal distribution in the analyzed subgroups was verified using the Kolmogorov–Smirnov test, after which Student’s *t*-test for independent samples was used to assess differences between the analyzed subgroups. When statistically significant differences were demonstrated, Cohen’s d test was performed for effect-size analysis. Subsequently, Pearson correlation analysis was performed between bone thickness at the POI and age in the overall group and in subgroups distinguished by sex. Statistical analysis was performed using Statistica 7.0 software (developed by StatSoft Inc., Tulsa, OK, USA).

## 3. Results

The results of nasal bone thickness measurements at points 1-5L and 1-5R are presented below in Table 1.

In the presented table, attention should be paid to the differences between the values 2L–5L, which amount to 0.91 and 2R–5R, which amount to 0.82.

Subsequently, statistical analysis was performed within the study group. First, the Kolmogorov–Smirnov test confirmed a normal distribution in all groups of variables; then, parametric analysis using Student’s *t*-test was performed and the data were entered into Table 2.

The above analysis showed statistically significant differences between subgroups distinguished by sex for parameters 1L (*p* < 0.01), 4L (*p* < 0.01) and 5L (*p* < 0.05), as well as 1R (*p* < 0.05) and boundary value of 5R (*p* = 0.05). At all analyzed points, nasal bone thickness values were significantly greater in men than in women.

In addition, effect-size analysis demonstrated a large effect for parameters 1L (0.85), 4L (0.81) and 1R (0.86), indicating strong differentiation between sexes in these locations. For parameters 5L and 5R, a medium effect was observed, which also confirms the significance of the differences, although with lower magnitude.

Next, to assess the effect of age on nasal bone thickness at the selected points, Pearson correlation analysis was applied (Table 3).

In the analysis performed, no statistically significant correlations between age and nasal bone thickness were found in any of the analyzed groups (overall group, men, women).

## 4. Discussion

As the number of rhinoseptoplasty procedures increases, the need for precise analysis of nasal shape also grows. Increasing importance is attached not only to knowledge of ethnic variants in nasal bone structure, but also to detailed knowledge of bony structures, such as the nasal bone and the piriform aperture, which have a substantial influence on the final appearance of the nose. Such data are useful not only in the clinical practice of otorhinolaryngologists and plastic surgeons for planning effective rhinoseptoplasty procedures, but also in anthropological research. From an anthropological standpoint, nasal bone length and piriform aperture width may be related to climatic conditions; in cold and dry climates, longer nasal bones and a narrower piriform aperture facilitate better warming and humidification of inhaled air [4,5,6].

Assessment of the bony structures of the nose may be performed using various diagnostic methods, such as physical examination, conventional radiography, 3D somatometric measurements or computed tomography with three-dimensional reconstruction. It should be emphasized, however, that palpation findings are characterized by considerable variability and may differ not only between individual physicians but even in the same examiner depending on the time of assessment, which makes it difficult to obtain objective and reproducible data.

Although mathematical models enabling precise anatomical measurements are available, their use in everyday clinical practice remains limited because of technical complexity and time requirements. In this context, computed tomography with 3D reconstruction is currently considered the most reliable method for obtaining accurate quantitative data on the nasal bone [2]. This technique is widely used not only in nasal plastic surgery, but also in the assessment of craniofacial growth and development in patients with congenital and acquired defects, as well as in planning complex reconstructions. It is particularly valuable in preoperative modeling of nasal structures, especially in cases requiring the use of bone or cartilage grafts, as well as materials such as acellular dermal matrix (ADM). This is especially important in the treatment of advanced deformities and defects, for example in saddle nose deformity [7,8].

The nasal bone is a key element determining the shape and aesthetics of the entire nose. Superiorly, it articulates with the frontal bone—laterally, with the frontal processes of the maxillae—and inferiorly, it contributes to the boundaries of the piriform aperture. Its size, shape and proportions show significant individual and ethnic variability. For example, in Asian populations the nose is typically wider and lower and the nasal bones are shorter, which distinguishes their anatomy from that observed in European populations. These differences are of major importance in planning and performing surgical procedures within the nose [2].

Osteotomy is a key component of rhinoseptoplasty that significantly influences the final nasal shape. Lateral osteotomy narrows the nasal base, whereas combining it with medial osteotomy improves control over nasal pyramid symmetry and contour. The location and course of the osteotomy line directly affect aesthetic outcomes and may influence structures such as the nasolacrimal duct and lateral nasal wall [9,10].

Although injury to the lacrimal system is uncommon when osteotomy is performed within safe anatomical zones, improper technique may result in transient epiphora or, rarely, permanent damage [11,12,13,14,15,16]. Postoperative tearing is usually caused by tissue edema and resolves spontaneously; Sachs et al. reported its occurrence in approximately 2% of rhinoplasty patients, with resolution within 6 months [15]. Permanent injury is mainly associated with incorrect surgical technique, particularly during subperiosteal dissection or osteotomy performed with a saw [2,15,16].

Attention should also be paid to one of the classical disadvantages of medial osteotomy, namely the possibility of palpable dorsal edges of the osteotomized bone and the presence of small irregularities or bony spurs. This phenomenon is referred to as rocker deformity and consists of characteristic displacement of the bony fragment, in which the cephalic edge deviates outward, whereas the caudal edge collapses excessively inward. It results from an excessively cephalic course of both the starting point and the direction of osteotomy, which promotes increased rotation of the mobilized bony fragment and exacerbates the deformity [17]. In lateral osteotomy, the risk of so-called staircase deformity increases with the degree of medialization of the bone and the higher the level of the fracture. In this technique, the “low-low-low” pattern is often used, in which the osteotomy is conducted from the lower parts of the lateral nasal wall medially up to the level of the medial canthus. This approach enables mobilization of a larger bony segment and theoretically reduces the risk of some irregularities; however, its important limitation is the possibility of creating a bony gap and a palpable edge of the maxillary bone [18]. In such clinical situations, double-level osteotomy is recommended; it is also used to correct asymmetry and excessive convexity of the lateral nasal wall, enabling more precise alignment of the bony structures. Technically, lateral osteotomy is performed using a 2-mm osteotome introduced subperiosteally and advanced in close contact with the bone surface. The procedure begins at the widest part of the lateral nasal wall, where “scraping” movements are used to create a controlled grooved osteotomy line. The osteotome is then moved alternately in the proximal and distal directions, allowing gradual medialization of the bony fragment [4,18].

Osteotomy in rhinoseptoplasty is a key step that enables correction of nasal deformities through precise cutting and repositioning of the nasal bones. The choice of the osteotomy site and the technique used depends on several factors, including nasal bone thickness. Differences in bone thickness influence the choice of osteotomy technique and the force required to perform it. Thicker bones in the lateral region may require the use of greater force or specialized instruments, whereas thinner medial bones are more prone to uncontrolled fractures, which requires a more delicate approach. Table 4 presents an overview of osteotomy techniques and their relationship to bone thickness.

### Significance of Anatomical Variations

The dimensions of the bony nasal vault significantly influence nasal shape and asymmetry; therefore, detailed anatomical assessment is essential before osteotomy. Key parameters in rhinoplasty planning include the dorsal aesthetic lines (DALs), lateral aesthetic lines (LALs), dorsal width (DW), and basal width (BW), which guide both preoperative analysis and postoperative outcome evaluation [2]. The continuity, symmetry, and proportions of these structures are critical for achieving natural and harmonious aesthetic results consistent with the individual anatomy of the patient. Ethnic differences in these parameters should also be considered, as they may influence surgical strategy and the extent of bony modification. Additionally, nasal bone thickness is an important factor affecting the choice of osteotomy technique, the force and direction of instrument guidance, fracture control and the risk of complications [5,19]. Because variations in bone thickness and resistance can influence bony fragment mobilization and surgical outcomes, their careful assessment is essential for operative planning. Successful osteotomy also requires meticulous planning, precise surgical technique, adherence to safe anatomical zones, and appropriate instrument selection, including conventional osteotomes or piezosurgical devices, to ensure predictable and atraumatic results [9,20].

Jae-Yong Jeong et al. [4] analyzed nasal osteotomy in Asian rhinoseptoplasty patients and highlighted important anatomical differences compared with Caucasians, including shorter and thinner nasal bones and a narrower keystone area, necessitating a more precise surgical approach. They identified the nasomaxillary transition zone (NMTZ) as a relatively safe pathway for lateral osteotomy and showed that osteotomes ≤ 3 mm (optimally 2 mm) reduce mucosal injury and postoperative edema. Maintaining at least a 2 mm distance between osteotomy lines was also recommended to improve bony fragment stability. Other anatomical studies have further demonstrated significant ethnic variation in nasal bone morphology. While Lang and Baumeister [20,21,22] proposed an eight-type classification for the German population, Hwang et al. [23] introduced a simplified five-type system for Korean patients, revealing markedly different distributions of nasal bone types. These findings emphasize the importance of ethnic differences in nasal anatomy and the need for individualized osteotomy planning and technique [6,23,24].

At the same time, the osteotomy technique itself is of key importance, with precision playing the primary role. It is recommended that cuts be made in anatomically safe designated zones while maintaining the proper sequence, medial osteotomy first and then lateral osteotomy, which allows controlled mobilization of the bony structures. In practice, both conventional osteotomes and piezosurgery-assisted techniques are used, enabling more atraumatic bone cutting. An important element of planning is also the assessment of nasal bone thickness, as it directly affects the selection of instruments and the force required to perform osteotomy, further emphasizing the importance of an individualized approach to each case.

Ofodile et al. [25], in turn, analyzed nasal bone morphometry, focusing on measurements of nasal bone length and piriform aperture width. Citardi et al. [26] expanded this issue to include assessment of nasal bone thickness and anatomical relationships between the rhinion and nasal tip. In their study, the authors performed preoperative computed tomography analysis in patients qualified for transsphenoidal hypophysectomy, excluding individuals with congenital craniofacial defects and those after previous nasal surgery. The assessment included, among other parameters, nasal bone thickness and the height of the nasal tip relative to the rhinion. They demonstrated that the mean bone thickness at the planned site of lateral osteotomy, corresponding to the region of the nasomaxillary suture in the axial section at the level of the rhinion, was 2.39 ± 0.68 mm (range 1.50–3.70 mm). In contrast, in the intermediate segment between the rhinion and the nasomaxillary suture, the thickness was significantly lower, with a mean of 1.18 ± 0.30 mm (range 0.50–1.00 mm). The mean difference between these locations was 1.21 mm, emphasizing significant variation in bone thickness along the planned osteotomy lines. For comparison, in the study by Seung Ho Lee et al. [4], much smaller differences in nasal bone thickness between the analyzed levels were observed, amounting to only approximately 0.28 mm, which indicates morphological differences depending on the studied population and potential clinical implications for planning and performing osteotomy [11]. In the authors’ study, attention should be paid to the differences between the values 2L–5L and 2R–5R, which amount to 0.91 and 0.82, respectively. The obtained discrepancies also indicate significant variation in dimensions within the analyzed measurement points, both on the left and right sides, which may reflect morphological heterogeneity of the examined structures (Table 1).

Interestingly, in the authors’ study, no statistically significant associations were demonstrated between measurement values and age, either in the analysis of the entire study group or in the subgroups of men and women (Table 3). This means that the assessed parameters showed no significant dependence on patient age. However, significantly lower nasal bone thickness was demonstrated in women compared with men (Table 2). It should be emphasized that the absence of a relationship between nasal bone thickness and age does not imply that bone remains biologically unchanged throughout life. While thickness may remain stable as a geometric parameter, bone quality and mechanical properties evolve with age due to ongoing remodeling and mineralization processes. Bone mineralization is an actively regulated process involving calcium and phosphate homeostasis, collagen matrix organization, alkaline phosphatase activity, and genetic factors such as PHEX, DMP1, and FGF23. Disturbances in these mechanisms can impair mineralization and lead to conditions such as osteomalacia. Thus, stable bone thickness does not necessarily reflect unchanged bone composition or strength. With age, qualitative changes in bone structure occur primarily, not necessarily marked “thinning.” Bone becomes more porous, the proportion of intraosseous spaces increases and mineral density decreases as a result of disturbances in the balance between formation and resorption processes and reduced effectiveness of hydroxyapatite mineralization. As a result, not only the quantity but above all the quality of the mineralized matrix changes, which weakens the biomechanical properties of bone, even though its overall dimensions may remain relatively stable [27]. As a result, bone loses elasticity and resistance to dynamic loads, becoming more brittle. In the context of osteotomy, this means that in older patients, despite nasal bone thickness similar to that of younger individuals, the force required to cut the bone may be lower and the fracture line may be less predictable. The bone becomes thinner and additionally changes its behavior under mechanical forces; uncontrolled cracks, microfractures and consequently irregular osteotomy lines occur more easily. Nasal bone mechanics are affected by changes in trabecular microarchitecture and potential processes related to physiological age-related osteopenia, which reduce the bone’s ability to dissipate energy evenly. In surgical practice, this translates into greater susceptibility to bone fragility despite the absence of significant differences in its thickness on imaging measurements. Conversely, the thinner nasal bone observed in women compared with men has important clinical significance in planning and performing osteotomy, even if it does not always translate into differences related to patient age alone. Lower thickness primarily means a shorter bony channel through which the osteotome passes, which may facilitate instrument penetration but at the same time increases the risk of uncontrolled bone breakage and formation of irregular fracture lines. In surgical practice, thinner bone in women may require less force during osteotomy, but at the same time it reduces the safety margin within which the direction and course of the fracture can be controlled. This means greater susceptibility to a so-called “greenstick fracture” or unintended propagation of a crack beyond the planned osteotomy line. In addition, a thinner bony structure is often associated with lower bone mass and potentially more delicate microarchitecture, which may affect the stability of the mobilized bony fragments obtained after osteotomy. As a result, although the procedure itself may technically be easier to initiate, it requires greater precision and control to avoid irregularities of the nasal dorsal contour and disturbances of symmetry. From the standpoint of surgical planning, these differences emphasize the importance of individualizing the osteotomy technique according to sex, because bony anatomy directly influences both the choice of instruments and the way the incision line is guided. It should also be emphasized that men have significantly longer nasal bones (Men: 30.61 ± 1.26 mm; Women: 29.01 ± 1.12 mm), which may additionally influence the choice of surgical techniques and osteotomy angles [28]. Particular clinical significance arises in the population of older female patients, in whom two factors overlap: the physiologically thinner nasal bone typical of women and age-related qualitative changes. Even if bone thickness on imaging measurements does not undergo further significant reduction with age, deterioration of its microstructure and mineralization occurs, increasing the brittleness of bone tissue. As a result, bone may be both thinner and less mechanically resistant, which has a direct impact on the course of osteotomy. Under such conditions, control over the fracture line decreases and the risk of unpredictable cracking, formation of irregular bony edges, or collapse of the nasal pyramid is greater. This applies especially in situations requiring greater medialization of bony fragments, where the forces acting on the structure are more complex. Therefore, in older women, gentler osteotomy, limiting the energy transmitted to the bone, and precise selection of instruments become particularly important. In clinical practice, this means the need for even greater control over the surgical technique, because even with normal bone thickness, its “mechanical quality” may be significantly reduced, increasing susceptibility to unwanted fractures and nasal contour irregularities [29,30,31]. Interestingly, despite the recognized anatomical differences between male and female nasal bones, evidence regarding their impact on osteotomy-related complications remains scarce. While the overall complication rate following nasal osteotomy has been reported to range between 4% and 18.8%, the available literature does not provide clear quantitative comparisons of complication rates between sexes [32,33].

The present study also confirmed significant variation in nasal bone thickness across its individual segments. The greatest thickness was observed in the upper nasal segment, whereas the smallest was found in the lower part. These findings are consistent with classical principles of osteotomy, according to which the lower segment of the nasal pyramid is more susceptible to controlled transection and mobilization, which is of major importance in planning the osteotomy line and selecting the surgical technique (Table 1). In addition, bone thickness was found to be greater medially than laterally, which has important practical significance because it suggests the need for caution in techniques requiring deeper penetration, such as medial osteotomies performed with a saw. Consequently, the obtained results emphasize the importance of individualized selection of instruments and an appropriate cutting force, which directly influences the safety and precision of the osteotomy performed. For example, according to Ofodile [25], the mean nasal bone length in Austrians was 3.02 mm (range 2.9–3.1 mm) and piriform aperture width was 2.16 mm (1.7–2.4 mm). Lang et al. [22] reported that in the German population, nasal bone length averaged 24.9 ± 3.2 mm and piriform aperture width 23.6 ± 1.8 mm. In Hwang’s studies [24], nasal bone length in Koreans averaged 25.9 ± 3.8 mm in men and 24.5 ± 3.7 mm in women, whereas piriform aperture width was 25.7 ± 1.7 mm and 25.4 ± 2.1 mm, respectively. In study [4], the nasal bone was shorter and the piriform aperture narrower than in Western populations. Nevertheless, 9 patients (12%) were found to have a long nasal bone, which may suggest that in their case, as Asian patients, percutaneous transverse osteotomy would have produced better results.

Overall, the results of the present study are consistent with data reported in previous publications and do not show significant deviations from them. It should be emphasized, however, that the frequency of individual morphological types and the obtained measurement values may differ depending on the size and characteristics of the study group, as well as on the adopted measurement methodology. Additional discrepancies may result from technical limitations of computed tomography, which in some cases may slightly underestimate the actual dimensions of anatomical structures. Despite these limitations, three-dimensional computed tomography remains a highly precise and objective method, clearly superior to conventional techniques of morphological assessment, while also constituting a valuable, noninvasive tool in preoperative diagnostics and surgical planning.

## 5. Conclusions

Understanding the relationship between nasal bone morphology and osteotomy technique is essential for achieving predictable, reproducible and safe outcomes in rhinoseptoplasty. The findings of the present study highlight the importance of accounting for individual variations in nasal bone thickness during preoperative planning as these anatomical differences may influence both the choice of osteotomy lines and the technical execution of the procedure. While the final aesthetic outcome is determined by multiple factors, including soft tissue characteristics and overall nasal proportions, a detailed understanding of the underlying bony framework remains fundamental to successful surgical planning.

The results obtained in this study may have important practical implications for rhinoseptoplasty. The morphometric data derived from three-dimensional CT analysis may assist plastic surgeons and otorhinolaryngologists in identifying anatomically favorable osteotomy sites and selecting the most appropriate surgical approach. Such knowledge may contribute to a more precise and safer surgical procedure while reducing the risk of technical difficulties and undesirable outcomes, including irregular bony contours, asymmetries or uncontrolled fracture propagation. In this context, osteotomy should be viewed not merely as a modification of the bony framework but as an integral component of a comprehensive surgical strategy aimed at optimizing both functional and aesthetic results. Although complications following osteotomy, such as temporary nasal obstruction, edema-related contour irregularities or, less commonly, injury to adjacent anatomical structures, may occur, they remain relatively uncommon when sound surgical principles are followed. Ultimately, successful outcomes depend on a combination of surgical expertise, meticulous preoperative planning and a thorough understanding of nasal anatomy. Consideration of individual anatomical variability, including sex- and age-related differences in nasal bone thickness, may facilitate a more personalized approach to treatment and contribute to improved surgical outcomes.

## Figures and Tables

**Table 1:** Comparison of nasal bone thickness at points 1-5L and 1-5R.

	Mean	SD	Mean Women	SD Women	%SD	Mean Men	SD Men	%SD
1L	7.27	2.27	6.29	2.00	0.32	8.07	2.19	0.27
2L	2.51	0.67	2.46	0.66	0.27	2.55	0.69	0.27
3L	2.37	0.57	2.31	0.59	0.26	2.41	0.57	0.24
4L	2.33	0.65	2.07	0.42	0.20	2.55	0.73	0.29
5L	1.60	0.20	1.52	0.17	0.11	1.66	0.20	0.12
1R	7.09	2.62	5.95	2.18	0.37	8.02	2.63	0.33
2R	2.55	0.62	2.63	0.58	0.22	2.49	0.66	0.26
3R	2.46	0.62	2.42	0.65	0.27	2.49	0.60	0.24
4R	2.39	0.78	2.22	0.57	0.26	2.52	0.91	0.36
5R	1.73	0.60	1.54	0.19	0.13	1.89	0.77	0.41

**Table 2:** Comparison of patient groups according to sex.

	Men K-S	Women K-S	Mean Men	Mean Women	t	*p*	Cohen’s d
1L	*p* > 0.05	*p* > 0.05	8.07	6.29	−2.68	0.0108	0.85
2L	*p* > 0.05	*p* > 0.05	2.55	2.46	−0.42	0.67	
3L	*p* > 0.05	*p* > 0.05	2.41	2.31	−0.54	0.4915	
4L	*p* > 0.05	*p* > 0.05	2.55	2.07	−2.6	0.013	0.81
5L	*p* > 0.05	*p* > 0.05	1.66	1.52	−2.37	0.01	0.75
1R	*p* > 0.05	*p* > 0.05	8.02	5.95	−2.4	0.01	0.86
2R	*p* > 0.05	*p* > 0.05	2.49	2.63	0.71	0.48	
3R	*p* > 0.05	*p* > 0.05	2.49	2.42	−0.32	0.37	
4R	*p* > 0.05	*p* > 0.05	2.52	2.22	−1.27	0.21	
5R	*p* > 0.05	*p* > 0.05	1.89	1.54	−2.05	0.05	0.62

**Table 3:** Comparison of correlations between patient age and nasal bone thickness at the POI in the analyzed groups (men, women, overall group).

Pearson: Age vs. Dimension						
	Men		Women		Overall	
	*p*	R	*p*	R	*p*	R
1L	>0.05	0.35	>0.05	−0.1197	>0.05	0.18
2L	>0.05	−0.13	>0.05	0.115	>0.05	0.03
3L	>0.05	−0.11	>0.05	−0.083	>0.05	−0.09
4L	>0.05	0.31	>0.05	−0.23	>0.05	0.17
5L	>0.05	0.2	>0.05	0.24	>0.05	0.22
1R	>0.05	0.3	>0.05	0.06	>0.05	0.22
2R	>0.05	−0.25	>0.05	0.06	>0.05	−0.15
3R	>0.05	0.13	>0.05	−0.32	>0.05	−0.04
4R	>0.05	0.32	>0.05	0.03	>0.05	0.25
5R	>0.05	0.22	>0.05	0.15	>0.05	0.2

**Table 4:** Osteotomy techniques and their relationship to bone thickness.

Technique	Description	Relevance to Bone Thickness
High-low-high osteotomy	The osteotomy begins high at the piriform aperture, descends low along the frontal process of the maxilla and then returns high toward the nasal bones. This approach preserves a triangular bony segment, reducing the risk of middle vault collapse.	Suitable for maintaining structural stability; commonly used regardless of bone thickness but particularly beneficial when preservation of support is critical
Perforating osteotomies	Performed using a series of small perforations along the planned osteotomy line, allowing a controlled fracture of the bone.	Particularly advantageous in regions with increased bone thickness, as it enables more precise and controlled bone division.

## Data Availability

The data presented in this study are available from the corresponding author upon reasonable request.
